# The Apelinergic System Immuno-Detection in the Abomasum and Duodenum of Sheep Grazing on Semi-Natural Pasture

**DOI:** 10.3390/ani11113173

**Published:** 2021-11-06

**Authors:** Elisa Palmioli, Cecilia Dall’Aglio, Michele Bellesi, Federico Maria Tardella, Sara Moscatelli, Paola Scocco, Francesca Mercati

**Affiliations:** 1 Department of FISSUF, PhD Course in “Ethics of Communication, Scientific Research and Technological Innovation” Medical-Health Curriculum, University of Perugia, Piazza G. Ermini, 1, 06123 Perugia, Italy; elisa.palmioli@studenti.unipg.it; 2 Department of Veterinary Medicine, University of Perugia, Via San Costanzo 4, 06126 Perugia, Italy; francesca.mercati@unipg.it; 3 School of Biosciences and Veterinary Medicine, University of Camerino, Via Pontoni 5, 62032 Camerino, Italy; michele.bellesi@unicam.it (M.B.); dtfederico.tardella@unicam.it (F.M.T.); s.moscatelli91@gmail.com (S.M.); paola.scocco@unicam.it (P.S.)

**Keywords:** apelin, apelin receptor, adipokines, digestive system, ovine, immunohistochemistry

## Abstract

**Simple Summary:**

The semi-natural pastures in the Apennines represent the feed source for ovine, whose grazing activity helps to preserve the grassland’s biodiversity. Summer drought stress decreases the grassland pastoral value and affects the morpho-functional features of sheep’s digestive systems. A better knowledge of the gastrointestinal system of sheep may contribute to guaranteeing their welfare, a prerequisite for the sustainability of livestock production. This study aimed to immune-localize the apelinergic system in the abomasum and duodenum of sheep grazing on semi-natural pasture during the spring–summer season and to compare its behavior among animal groups fed with or without supplementation. The apelinergic system, composed of apelin and its receptor, is involved in foodintake and the secretion and absorption activities of the digestive apparatus. Apelinergic system molecules were localized at the abomasum lining epithelium and fundic glands level and at the duodenum lining and crypt epithelium, in addition to the neuroendocrine cells. Variations in reactivity were observed in the different feed groups; feed supplementation seemed to maintain the functionality of the apelinergic system in the organs near the status related to the better pasture phase, suggesting that it may be a suitable solution able to counteract the harmful effects of summer drought stress.

**Abstract:**

Apelin (APLN) is an adipokine mainly produced by adipose tissue and related to an individual’s nutritional status as well as digestive apparatus functions. In this work, APLN and its receptor (APLNR) were investigated, by immunohistochemistry, in the abomasum and duodenum of 15 Comisana × Appenninica adult sheep reared in a semi-natural pasture. Organ samples were collected after maximum pasture flowering (M × F group) and after maximum pasture dryness (M × D group); the experimental group (E × p group) received a feed supplementation of 600 grams/day/head of barley and corn in addition to M × D group feeding. APLN and APLNR were identified in the lining epithelium and the fundic gland chief cells of the abomasum. APLNR was observed in the lining epithelium, in the crypts and the serotonin secreting cells of the duodenum. Similar reactivity was observed between the M × F and E × p groups, while the M × D group showed a lower intensity of immunostaining for both APLN and APLNR in all positive structures but the duodenal serotonin neuroendocrine cells. Hence, our findings show that the E × p group presents a picture quite overlapped with M × F and suggest that food supplementation has a maintaining effect on the apelinergic system expression in the investigated digestive tracts of the sheep.

## 1. Introduction

Adipose tissue acts as an organ producer of adipokines [1], such as apelin (APLN). APLN is a recently discovered regulatory peptide, initially isolated from bovine stomach extracts [2] and identified as an endogenous ligand of its receptor, APLNR, which together constitute the apelinergic system [3]. Recent studies reported the presence of APLN in adipocytes of several species [4], where the peptide is involved in energy metabolism. A sort of feedback takes placebetween APLN and insulin since this adipokine regulates the secretion of insulin through the APLNR expression in pancreatic islet β-cells [5]. In particular, it inhibits glucose-stimulated insulin secretion in mice and in isolated islets of Langerhans [6,7]. APLN expression in adipose tissue, as well as circulating APLN concentration, depend upon the nutritional status. In fact, APLN expression and plasmatic level are inhibited by fasting and recovered after refeeding [8]. APLN expression increases during adipogenesis so that the APLN plasmatic level positively correlates with body mass index in humans [9] and it is increased further in obese patients [8,10].

APLN and its receptor have a widespread distribution in both the central nervous system and the periphery. So, the apelinergic system takes action in the physiology of several organs and systems, such as the brain, heart, lung, liver and digestive and reproductive apparatus of humans, rats, bovines, swine and ovine [3,6,11,12,13,14].

APLN and APLNR expression has been detected in the gastrointestinal tract of rodents and humans, with higher levels in the stomach than in the small intestine [15,16,17,18,19]. It has been suggested that APLN plays a role in the regulation of feeding behavior and fluid intake [20,21,22], digestive motility, absorption and digestive secretions [16].

To date, there have not been any trials about the localization and role of the APLN system in the abomasum and duodenum of sheep. The abomasum represents the glandular stomach of ruminants, and its function is very important for their digestive efficiency. This organ produces gastric juices, kills forestomach microorganisms and digests both animal and plant proteins. For grazing ruminants, to which the ovine species belongs [23], the abomasum is fundamental for the denaturation of hemicelluloses by hydrochloric acid. Ovine diet, in fact, is based mainly on hemicellulose pabular species; the denaturation of hemicelluloses is mandatory for their following digestion in the distal fermentation chambers, consisting of the cecum and the proximal loop of the colon [24]. Accordingly, the functionality of the gastrointestinal tract has remarkable importance since its impairment affects the animal’s metabolism and so its wellness [25].

Based on these considerations, this study aimed to localize the apelinergic system in the abomasum and duodenum of sheep grazing on semi-natural pasture in the Central Italian Apennines. The grasslands represent a feed source for grazers and, at the same time, grazing activity helps to preserve natural grassland biodiversity [26]. Due to climatic change, which enhances summer drought stress, the moment of maximum flowering of the pasture tends to be anticipated and the period between the maximum flowering and the maximum dryness of the pasture to be reduced [27]. Summer aridity decreases the grassland pastoral value, above all in the period from the maximum pasture flowering to the maximum pasture dryness, consequently affecting the morpho-functional features and physiology of the digestive system of animals [27,28]. Hence, it is necessary to find suitable solutions able to counteract the harmful effects of summer drought stress on pastoral value. A better understanding of the gastrointestinal system of sheep, the most reared species in the Italian Apennine, can contribute to improving the management of these animals and safeguarding their welfare, which is a prerequisite to optimize the efficiency and sustainability of livestock production.

## 2. Materials and Methods

### 2.1. Animal Recruiting and Sample Collection

The study was performed on a flock of 15 Comisana × Appenninica 3-year-old female sheep for a period of three months [12]. The sheep were free to graze on the pasture from June to the maximum pasture flowering (early July) feeding on fresh forage; after this period, 5 subjects were slaughtered (M × F group). Then, sheep were divided into two homogeneous groups as regards age, reproductive performance and body condition score (BCS), as previously described (Mercati et al., 2018), throughout the period between the maximum pasture flowering and the maximum pasture dryness (early September). During this period, 5 subjects (M × D group) were grazing on pasture and feeding only on fresh forage, while the other ones, namely the experimental group (E × p group), also received a feed supplementation of 600 grams/day/head of barley and corn (1:1). The animals of the E × p group received food supplementation all together, before and after the daily period of stay on the pasture. The nutritional value of the feed is described in Table S1.

For use in investigations, samples about 1 cm^2^ wide were collected from the fundus of the abomasum, where the fundic glands or proper gastric glands reside, and the duodenum (approximately 10 cm from pyloric sphincter) [29] and were quickly fixed as described below.

The animals, intended for human consumption, were slaughtered at the abattoir in accordance with Art. 29 of the Council Regulation (EC) No. 1099/2009 on the protection of animals at the time of killing under law n.333/98 (Council Directive 93/119/EC of 22 December 1993) as specified by Annex C of Section II.

### 2.2. Morphological Staining and Immunohistochemistry

Samples were quickly fixed by immersion in 10% formaldehyde solution in phosphate-buffered saline(PBS 0.1 M, pH 7.4) for 36 h and processed until the paraffin wax embedding step. Fixed samples were dehydrated in a series of ethanol solutions with increasing concentrations and cleared in xylene to be included in paraffin wax. Five μm thick sections were cut, mounted on poly-L-lysine-coated glass slides and air dried at 37 °C. Hematoxylin–eosin staining was first performed on all specimens to carry out a morphological evaluation and to exclude pathologies.

Immunohistochemistry was performed on all samples as follows [30]: sections were dewaxed in xylene and hydrated through a series of ethanol concentrations and until to distilled water. Sections were microwaved for three 5 min cycles at 750 W in citrate buffer (pH 6.0) for antigen retrieval and treated for 10 min with a 3% hydrogen peroxide solution for endogenous peroxidase inhibition. Non-specific bindings were avoided with a 30 min application of normal goat serum.

For the immunohistochemical reaction, sections were incubated overnight at room temperature (RT) with a 1:200 rabbit polyclonal anti-APLN antibody (Novus Biological, Littleton, CO, USA), a 1:400 rabbit polyclonal anti-APLNR antibody (Abnova, Taipei City, Taiwan) and a 1:150 rabbit polyclonal anti-APLNR antibody (Novus Biological, Littleton, CO, USA). The dilution used for each primary antibody was the best in order to obtain the intensity of the signal and reduce the background among other dilutions tested in a range from 1:50 and 1:500. On the second day, sections were incubated with a 1:200 diluted goat anti-rabbit biotin-conjugated secondary antibody and the site of immunological reaction was detected with avidin–biotin complex (VectastainElite ABC Kit) and revealed with DAB chromogen (DAB substrate kit). Sections were also counterstained with hematoxylin. All steps were performed at room temperature and the slides were incubated in a humid chamber. The sections were washed with PBS between all incubation steps, except after normal serum.

Negative control sections were incubated with normal rabbit IgG (Novus Biological, Littleton, CO, USA), omitting the primary antibody. A sheep uterus was used as a positive control for the apelinergic system [13]. A photomicroscope (Nikon Eclipse E800, Nikon Corp., Tokyo, Japan) connected to a digital camera (Nikon Dxm 1200 digital camera) was used to observe all sections.

The intensity of the staining for APLN and APLNR was graded in arbitrary units [31] as follows: absent (0), weak (0.5), moderate (1), strong (2) and very strong (3). Three independent observers performed staining evaluation; each observer evaluated three randomly chosen fields into each slide, establishing the mean intensity values.

### 2.3. Double-Label Immunohistochemistry

A double-label localization of APLN and APLNR with serotonin in the abomasum was performed in accordance with a previously described method [32]. Briefly, APLN or APLNR-containing cells stained with DAB were treated with an Avidin/Biotin blocking kit (Vector), incubated with normal horse serum for 30 min and incubated overnight with a 1:50 mouse anti-serotonin antibody (M0758, DakoCytomation, Glostrup, Denmark). After incubation with a 1:200 horse anti-mouse biotin-conjugated secondary antibody, detection was performed with ABC complex (Vector) and SG (Vector) as a chromogen. 

An immunofluorescent double-label localization of APLNR with serotonin was performed on samples of duodenum fixed in 4% paraformaldehyde solution in phosphate-buffered saline (PBS). The sections were washed with PBS for three 5 min cycles. Then, they were incubated with normal goat serum for 1 hour and a half and incubated overnight with a 1:500 rabbit anti-APLNR antibody (Novus Biological, Littleton, CO, USA) and with a 1:500 mouse anti-serotonin antibody (M0758, DakoCytomation, Glostrup, Denmark). The next day, after being washedwith PBS for four 5 min cycles, the sections were incubated with normal goat serum for 30 min and reacted with a 1:1000 goat anti-rabbit secondary antibody (Alexa Fluor 488, Invitrogen, Thermo Fisher Scientific) and with a 1:1000 goat anti-mouse secondary antibody (Alexa Fluor 594, Invitrogen, Thermo Fisher Scientific) for 1 hour and a half. Later, the sections were washed with PBS for two 15 min cycles and finally counterstained with DAPI solution (Invitrogen, Thermo Fisher Scientific).

### 2.4. Statistical Analysis

To test the null hypothesis of no difference among different experimental groups for each immunohistochemical treatment, we performed Kruskal–Wallistests as the variables did not satisfy the assumptions for parametric tests (normality and homogeneity of variance were tested using the Shapiro–Wilk test and the Levene test, respectively). We ran pairwise comparisons using the Wilcoxon rank sum test to identify which groups were significantly different from each other. A Holm correction for multiple comparisons was used to avoid a type I error.

Statistical elaborations were performed using the R version 3.5.3 [33], the stats R-package, version 3.5.3 (shapiro.test, kruskal.test, wilcox.test) and the car R-package, version 3.0-2 (leveneTest function).

## 3. Results

**Figure 1:**
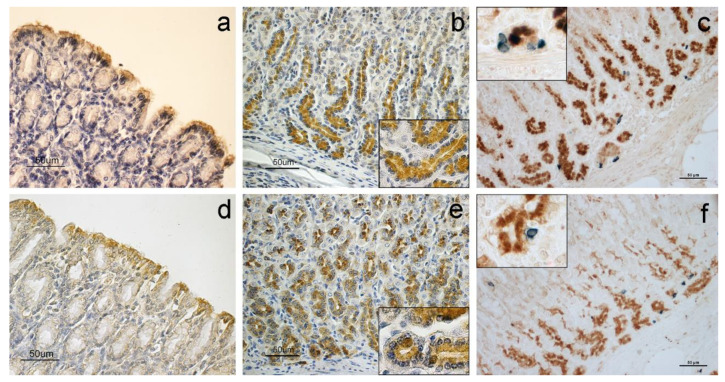
Immunostaining for APLN, APLNR and serotonin. Positivity to APLN (**a**) and APLNR (**d**) are localized in the epithelium of the abomasum. Fundic glands are positive to APLN (**b**) and APLNR (**e**); each inset shows a magnification of the corresponding image. Double-label immunohistochemistry shows serotonin-positive neuroendocrine cells (blue stained) that are distinct from APLN- (**c**) and APLNR- (**f**) positive cells (brown stained).

The immunohistochemical evaluation detected the presence of anapelinergic system in all analyzed samples of the abomasum, revealing both APLN and APLNR antibody binding sites. The positive cells were found in the mucous layer, especially in the lining epithelium (Figure 1a,d), and in the lower region of fundic glands (Figure 1b,e) where the immunohistochemical staining was appreciated in the chief cell cytoplasm and supranuclear region (see Figure 1b,e insets). In contrast, the parietal cells and neck cells were negative to the apelinergic system.

Double-label immunohistochemistry showed that neuroendocrine cells, positive to serotonin, did not stain with APLN nor APLNR (Figure 1c,f).

The negative control sections did not show any positive reaction.

**Figure 2:**
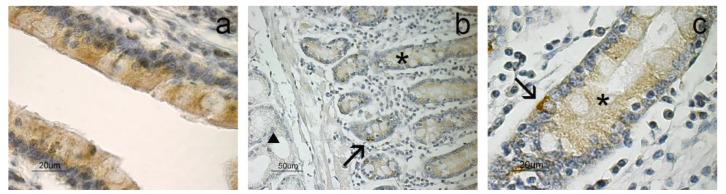
Immunostaining for APLNR in the duodenum. Positivity to APLNR is shown in the epithelium (**a**), intestinal crypts (*) and neuroendocrine cells (arrows; (**b**,**c**)), while duodenal glands (arrowhead) appear negative (**b**). A higher magnification of an intestinal crypt (*) with a positive neuroendocrine cell (arrow) is shown (**c**).

In the duodenum, positivity for APLN was not detected, while APLNR staining was observed in the lining epithelium (Figure 2a), intestinal crypts (Figure 2b) and neuroendocrine cells (Figure 2c). The duodenal glands located in the submucosal layer were negative (Figure 2b). The APLNR staining in the intestinal crypts and epithelium seemed lighter than in the neuroendocrine cells.

**Figure 3:**
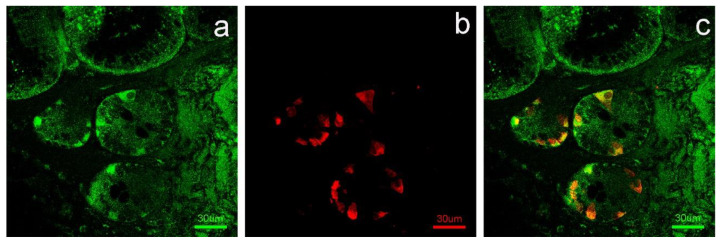
Immunofluorescence for APLNR ((**a**), green) and serotonin ((**b**), red) in the intestinal crypts of the duodenum. Colocalization of APLNR and serotonin is showed in neuroendocrine cells ((**c**), merge).

Double-label immunofluorescence showed neuroendocrine cells, positive to serotonin, stained with APLNR (Figure 3).

The results of semiquantitative evaluation of immunopositivity, expressed as mean values, aresummarized in Table 1.

Comparing the staining intensity among the three different animal groups, it can be observed that the M × F group and E × p group samples generally showed similar responses, while the M × D group samples showed different, often lower, reactivity.

In the abomasum, the fundic glands showed significantly stronger reactivity for both APLN and APLNR in M × F and E × p groups with respect to M × D sample positivity; a similar trend was observed for APLNR positivity of the lining epithelium.

In the duodenum, both lining epithelium and intestinal crypt reactivities of M × F and E × p groups showed significant differences with respect to M × D sample positivity, which was lower than in the other groups. Neuroendocrine cells showed the highest reactivity in M × D group samples, which was significantly different from M × F ones.

The significance of differences among the diet groups for each immunohistochemical treatment is shown in Table 2.

## 4. Discussion

This research describes the presence and the localization of the apelinergic system in the abomasum and duodenum of sheep grazing on semi-natural pasture and evidences differences related to diet. By immunohistochemistry, APLN and APNLR were observed in the mucosa layer of the abomasum and duodenum. 

As far as the abomasum is concerned, molecules were identified in the lining epithelium and in the fundic glands, in which the cells of the lower third of the glands showed abundant APLN and APLNR localization, while the neck cells were negative. APLN was initially characterized by Tatemoto et al. [2] from bovine stomach extracts, while its localization was previously described in other species, including rats and humans [18], with which our results partially agree. Similarly, APLNR had already been localized in the rat epithelial lining [19] and rabbit enteroendocrine cells [34] of the stomach. 

The positive cells of the fundic glands of the abomasum have been labeled as chief cells based on their morphological characteristics and localization [17,18,35]. In addition, abundant APLN-positive cells were previously identified in rat and human stomach–oxyntic epithelium [17,18]. Parietal cells,whichare strongly acidophilic and mainly located in the body region of fundic glands [29,36], clearly appeared negative to both APLN and APLNR in the glandular structures. However, Susaki et al. [17] observed both APLN-positive and -negative parietal cells in rat stomachs. Studies performed on rats indicate that APLN is produced in both gastric exocrine and endocrine cells [17] since enteroendocrine cells producing chromogranin A co-localized with some APLN-positive cells. However, we observed that in sheep, serotonin-secreting enteroendocrine cells did not co-localize with APLN and its receptor. These differences may be due to the species investigated or to different markers used to identify neuroendocrine cells; indeed, ultrastructural studies have identified at least six distinct cell types in the gastric endocrine cell population with different secretory products, including serotonin [37].

APLN immunohistochemical staining was detected in the cytoplasm; the localization of APLN in both the supranuclear and apical region suggests its secretion in the lumen of the gastric glands and, hence, in the lumen of the stomach through an exocrine mechanism as already supposed in other species [18] and for other gastric peptides such as leptin [38]. In addition to the stomach, the exocrine action had already been hypothesized for breast tissue because APLN increases in the lactation period, and it is abundantly present in colostrum [39].

Our findings show that in the abomasum, APLN and APLNR are localized on the same structures and cells; for this reason, it is possible to hypothesize an autocrine action of the APLN on the chief cells, likely aimed at regulating epithelial and principal cell turnover in adult animals. In vitro studies attested the ability of APLN to stimulate the proliferation of human stomach epithelial cells [18].

As far as the duodenum is concerned, APLN was not evidenced, while APLNR was observed in the mucosa layer. Previous studies demonstrated that APLN is expressed in rat duodenum, even if they failed to observe the protein by immunohistochemistry [18]. We observed APLNR in the lining epithelium, intestinal crypts and serotonin-positive neuroendocrine cells. APLNR immunostaining was previously observed in the duodenum of the developing and adult rat [19]. APLNR staining in the epithelial lining and intestinal crypts suggests that APLN may be implicated in the epithelial proliferation [19]; indeed, Han et al. [40] demonstrated that APLN can stimulate intestinal epithelial proliferation. 

In the mouse and rat intestinal STC-1 enteroendocrine cell line, apelin-13 stimulated CCK [18] and incretin GLP-1 [41] secretion. Previous authors hypothesized that the hormones produced by neuroendocrine cells of the intestine may mediate the enteric and/or systemic action of APLN [41]. In the sheep, we observed that serotonin-positive cells located in the mucous layer of the duodenum showed intense immunostaining to APLNR, suggesting that these cells may represent the specific binding sites for the APLN secreted in the abomasum. The same hypothesis can be applied to the APLNR-positive cells observed in the epithelial lining and intestinal crypts. Indeed, Wang et al. [18] showed that APLN, abundantly observed in the abomasum, can be secreted into the lumen of the organ and reach the duodenal lumen.

We observed variations in the intensity of the immunopositivity for APLN and APLNR among the different sheep groups, likely reflecting the expression of the corresponding antigens. The comparison among the three animal groups showed a similar reactivity between the M × F and E × p groups. The M × D group showed a different and lower reactivity, with the exception of neuroendocrine cells. 

Regarding sheep feed, the M × F group fed on a fresh pasture at the maximum flowering phase, when forage had a high content of proteins and water and a low content of fibers. In contrast, the M × D group grazed on a pasture during the dryness phase, when forage contained a high amount offiber; moreover, some fibers were represented by indigestible lignin [42]. Sheep of the E × p group, in addition to being fed with the same forage as the M × D group, received a feed supplementation of barley and corn, particularly enhancing the protein intake.

Feed supplementation seems to have a maintaining effect on the apelinergic system expression. In fact, there was no difference in the response to the immuno-treatments with both APLN and APLNR antibodies between the M × F and E × p groups, while the M × D group showed significantly lower levels of APLN in the fundic glands, as well as of APLNR in the epithelial lining and fundic glands of the abomasum, and in the epithelial lining and intestinal crypts of the duodenum. On the contrary, as regards duodenal neuroendocrine cells, a significantly higher presence of APLNR binding sites was observed in the M × D group than in the M × F one; the E × p group did not show any significant difference when compared with both M × F and M × D groups. 

The above considerations allow us to think that a more nutritious diet results in the higher production of the apelinergic system molecules at the peripheral level, in this case referring to the abomasum and duodenum. Previous research demonstrated that there was not an APLN blood level modification during the trial, even if the apelinergic system had differentiated local effects on mammary glands and reproductive apparatus [12,13]. Similarly, it was observed that the adipokine leptin also did not show variation at blood level in the same animals as the trial [43]. 

The higher APLN level in the abomasum of the M × F and E × p groups leads us to speculate that APLN could exert an autocrine action on epithelial cells that may positively affect the mucous layer turnover. On the other hand, we observed a significantly higher APLNR-positive site presence in the M × F and E × p groups when compared with the M × D one, in both lining and crypt epithelial cells of the duodenum. At these sites, through the passage of the abomasum content, the APLN secreted by the fundic glands in significantly higher amounts in the better fed groups could have the same effect. Indeed, previous studies demonstrated that APLN stimulates the proliferation of stomach and intestinal epithelial cells in humans and rodents [18,40].

Finally, as regards duodenal neuroendocrine serotonin-positive cells, we observed a decreasing trend in the response to the APLNR antibody from M × D to E × p to M × F groups, with significant differences between the M × F and M × D samples. The effects of serotonin on intestinal activity regulation are particularly wide [44]; among these, serotonin regulates the absorption of nutrients [45,46] and the secretion of water, mucus and electrolytes [47,48,49] in the intestinal tract. So, the presence of APLNR at this site level can be explained with a positive effect of APLN on serotonin production. In addition to serotonin, APLN stimulates other signaling molecules secreted by enteroendocrine cells, including cholecystokinin and glucagon-like peptide 1 [18,41,50], and influences duodenal bicarbonate secretion. 

Our findings seem to suggest that APLNR is produced by serotonin-positive cells in a greater amount in an attempt to better intercept the APLN molecules produced in a loweramount when a worse feed is available.

## 5. Conclusions

Our findings support the idea that an inexpensive and easily manageable feed supplementation could represent one of the suitable solutions to counteract the harmful effects of summer drought stress on pastoral value in Central Italian Apennine pastoral ecosystems. As regards the apelinergic system, the E × p group shows a picture quite overlapped with that of the M × F one, avoiding the potential decrease in the digestive efficiency shown by the M × D group, as well as previously observed in mammary glands.

## Figures and Tables

**Table 1:** Responses by immunoreactive structures to APLN and APLNR detection treatments in the three different animal groups, expressed as mean intensity values.

Organs	Searched Molecules	Immunoreactive Structures	M × F	E × p	M × D
Abomasum	APLN	Lining epithelium	1.15	1.35	1
		Fundic glands	1.98	2.13	1.18
	APLNR	Lining epithelium	0.85	0.87	0.48
		Fundic glands	1.18	1.2	0.31
Duodenum	APLNR	Lining epithelium	1.87	1.79	1.03
		Intestinal crypts	1.5	1.66	0.84
		Neuroendocrine cells	1.7	2.33	2.7

**Table 2:** Statistical significance of differences (*p* < 0.01) for each histochemical treatment among different animal groups, as performed by Kruskal–Wallis tests, and respective pairwise comparisons, as performed by Wilcoxon rank sum tests. *p*-values were adjusted for multiple testing using the Holm correction. Significant values are indicated in bold.

Organs	Searched Molecules	Immunoreactive Structures	Kruskal–WallisTest	Wilcoxon Rank Sum Test		
				M × F vs. E × p	M × F vs. M × D	E × p vs. M × D
Abomasum	APLN	Lining epithelium	0.1567	0.41980	0.41980	0.22803
		Fundic glands	**1.439 × 10^−7^**	0.0702	**7.92 × 10^−6^**	**7.92 × 10^−6^**
	APLNR	Lining epithelium	**0.000352**	0.8329	**0.00171**	**0.00171**
		Fundic glands	**3.129 × 10^−7^**	0.7981	**8.277 × 10^−6^**	**8.277 × 10^−6^**
Duodenum	APLNR	Lining epithelium	**2.827 × 10^−7^**	0.4076	**8.517 × 10^−6^**	**8.517 × 10^−6^**
		Intestinal crypts	**1.716 × 10^−6^**	0.2282	**1.453 × 10^−4^**	**8.829 × 10^−6^**
		Neuroendocrine cells	**0.002526**	0.0601	**0.0080**	0.0601

## Data Availability

The data are stored in the personal archives of the Authors.

## Supplementary Materials

The following are available online at https://www.mdpi.com/article/10.3390/ani11113173/s1, Table S1: Composition of the feed supplementation (%).
