# Supplementary material for: The Apelinergic System Immuno-Detection in the Abomasum and Duodenum of Sheep Grazing on Semi-Natural Pasture
Source: Animals (Basel). 2021 Nov 6;11(11):3173. doi: 10.3390/ani11113173 (PMC8614516; doi:10.3390/ani11113173)
Supplement: Supplementary file 1 [file animals-11-03173-s001.zip › animals-1448947-supplementary.pdf]

The apelinergic system immuno-detection in the abomasum and duodenum of sheep grazing on semi-natural pasture

Elisa Palmioli, Cecilia Dall'Aglio, Michele Bellesi, Federico Maria Tardella, Sara Moscatelli, Paola Scocco, Francesca Mercati

Table S1. Composition of the feed supplementation (%).

Dry matter	88.5
Crude protein	10
Lysine	0.3
Methionine	0.16
Tryptophan	0.09
Fat	3.0
Crude fibre	3.7
Ash	1.8
Ca	0.03
P	0.31
Starch	57.5
Free sugars	2.1
NDF (% DM)	12.5
Lignin	1.3
UFL (energy)	1.05
